# Electrolyte Coatings for High Adhesion Interfaces in Solid-State Batteries from First Principles

**DOI:** 10.1021/acsami.3c04452

**Published:** 2023-09-08

**Authors:** Brandi Ransom, Akash Ramdas, Eder Lomeli, Jad Fidawi, Austin Sendek, Tom Devereaux, Evan J. Reed, Peter Schindler

**Affiliations:** † Department of Materials Science and Engineering, Stanford University, Stanford, California 94305, United States; ‡ Aionics, Inc., Palo Alto, California 94301, United States; § Stanford Institute for Materials and Energy Sciences, Stanford University, Stanford, California 94305, United States; ∥ Department of Mechanical and Industrial Engineering, Northeastern University, Boston, Massachusetts 02115, United States

**Keywords:** adhesion, solid state, first-principles, lithium, interface

## Abstract

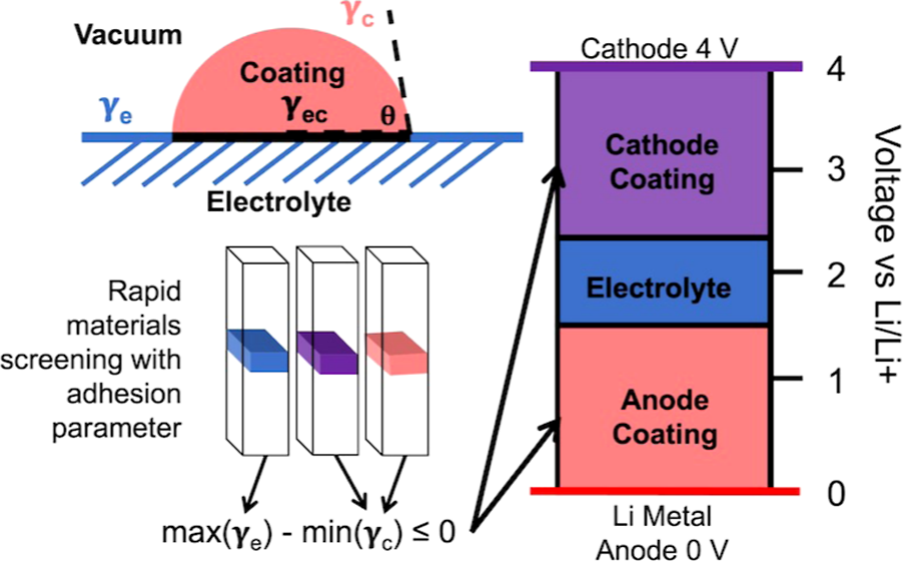

We introduce an adhesion parameter that enables rapid screening for materials interfaces with high adhesion. This parameter is obtained by density functional theory calculations of individual single-material slabs rather than slabs consisting of combinations of two materials, eliminating the need to calculate all configurations of a prohibitively vast space of possible interface configurations. Cleavage energy calculations are used as an upper bound for electrolyte and coating energies and implemented in an adapted contact angle equation to derive the adhesion parameter. In addition to good adhesion, we impose further constraints in electrochemical stability window, abundance, bulk reactivity, and stability to screen for coating materials for next-generation solid-state batteries. Good adhesion is critical in combating delamination and resistance to lithium diffusivity in solid-state batteries. Here, we identify several promising coating candidates for the Li_7_La_3_Zr_2_O_12_ and sulfide electrolyte systems including the previously investigated electrode coating materials LiAlSiO_4_ and Li_5_AlO_8_, making them especially attractive for experimental optimization and commercialization.

## 1 Introduction

The battery market is experiencing incredible growth with projections showing no signs of slowing down.^1^ Electric vehicles, mobile electronics, grid-scale renewable energy farms, and implantable medical devices are incredibly diverse examples of devices relying on batteries. As electrification becomes the norm, these various utilities will require tailored performance optimization of cycle life, operating voltage, and power. In many previous works, battery materials have been investigated for maximizing these properties among others and the methods developed therein could be repurposed to target specific values, not just the maxima.^2−4^ The vast growth of the battery market would also be easier to sustain with a more diverse set of battery materials. The detrimental environmental and social effects from the dependence on cobalt for Li–Co–O cathodes have become apparent.^5^ A few solid-state material optimizations have begun to take this into account when presenting novel materials for battery applications, showing the viable possibilities of high-performance solid-state materials with comparable performance to liquid batteries.^6^ These novel materials searches are pushing the solid-state field forward in optimization techniques and our understanding of structure-electrochemical performance relationships; however, novel materials alone are not enough to upend the liquid battery industry.

Solid-state battery success will also likely depend on a Li metal anode to become commercially viable in its energy density performance. Due to lithium metal’s high reactivity, there are very few materials which can come into contact with Li metal without reacting, typically lithium binary salts, which have insufficient ionic conductivities.^7^ Additionally, ionic conductivity and thermodynamic stability are bulk properties, which are insufficient to predict the performance of an operational battery cell because of the critical impacts of interfacial interactions on performance. The key to success in liquid batteries is the self-passivating solid electrolyte interphase layer between the electrolyte and the electrodes, a concept which has only begun to be investigated in the solid-state field. Further development toward finding solid materials which are not reactive at the interface is a foundational constraint for any battery, but it does not account for the operational voltage or interfacial resistance requirements.^8^ Coatings are highlighted as the solution to meeting both requirements, allowing a much broader set of electrolyte/electrode materials to be combined for successful batteries.

Computationally generated thermodynamic data has been readily used for electrochemical compatibility between solid electrolytes and electrodes, a necessary first step for describing the stability constraints on chemistries of an all solid-state battery.^9^ Coatings must have electrochemical stability windows (ESWs) large enough to overlap the full operational range of the electrode; however, two materials that are chemically nonreactive and are stable within the same voltage ranges can face large interfacial resistances.^10^ Solutions to interfacial resistance involve maximizing interfacial contact between materials, which has been explored on the macroscale with various syntheses and assembly techniques.^11^

Strong adhesion leads to longer mechanical life in batteries, as it helps maintain a well-contacted interface throughout cycling and volume changes of battery components. This was shown experimentally by applying pressure to cells, forcing interfaces to be better wetted, or adhered.^12^ In solid-state batteries, increasing the area of contact between materials is critical to taking advantage of the bulk ionic conductivity of promising Li–P–S and Li–La–Zr–O systems. These systems are also promising in that the Li–P–S system has previously been investigated for high interfacial contact due to its low Young’s modulus, and Li_7_La_3_Zr_2_O_12_ has shown the most promising stability in the field.^13−15^ However, to realize these compounds to have the same 4 Volt ESW we expect from liquid electrolytes, we would need solid coatings between these electrolytes and their electrodes.

Adhesion strength has been experimentally characterized by contact angle measurements of a drop of liquid on a solid surface,^16^ but it is challenging to perform this experiment with ceramic materials typical of solid-state battery components. Instead, for thin-film coatings, a laser-induced spallation technique has shown to be a more accurate noncontact adhesion measurement method.^17^ There have also been recent advances in first-principles workflows to determine the adhesion strength by calculating the potential energy surface of interfaces using density functional theory, but has been limited to elemental crystals and simple binary compounds.^18,19^

This work focuses on the development of an effective approximation of adhesion between solid materials and uses the screening of coatings for solid-state batteries as the test canvas due to the breadth of materials relevant to the space and the rapid progression of the technology. Specifically, we explore coatings that would be stable with the ideal Li metal anode, well-researched cathodes, and the promising electrolyte systems (LLXO, X = Zr or Ta), Li–P–S (LPS), and Li–B–S (LBS) (defined in Section 2.1). We prioritize common materials in our screening to show the chemically diverse and viable options for all solid-state battery chemistries. The approximation allows for extremely fast narrowing of the viable candidate space for any problem in which adhesion would benefit the end goal. The continuous nature of the adhesion parameter allows for ranking of all materials of interest for direct comparison. High-adhesion coatings with interfacial compatibility provide the physical bridge to realize higher voltage cathodes, metal anodes, and better performing interfaces for next-generation solid-state batteries.

The remainder of this paper proceeds as follows. Sections 2 and 3 establish the assumptions and boundaries of our approximation of the calculated adhesion parameter, outline the data used in our screening process, and describe each of the solid electrolyte systems chosen. Section 4 establishes the screening progression and overall viability of coatings for each of the solid electrolyte systems.

## 2 Materials and Methods

The methods developed throughout this work were applied specifically to solid-state interfaces of battery-compliant materials. This is only an application of the adhesion parameter, and the methods can be applied to other solid–solid interface use cases.

### 2.1 Data Description

Our pool of data originates from the materials project database (extracted: March 2022), which contains structures and density functional theory (DFT)-based data regarding 19,481 lithium containing compounds.^20^ These materials were the starting candidates for coatings which could be applied to the cathode or anode side of a solid electrolyte. Three specific chemical systems (LBS, LPS, and LLXO) were chosen as sample solid electrolytes for this work. These were chosen because of the increased focus on optimization of these compounds for commercialization in the literature, to show that the materials selected as “best candidates” in this work may be worth further pursuit. The specific electrolytes’ chemical formulas and their materials project ID numbers are listed here.
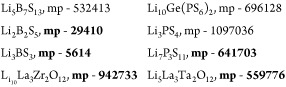


Materials in bold have experimentally validated structures. Materials which have not been experimentally verified by the materials project database were matched to literature based on space group.

### 2.2 Screening Metrics for Coating-Electrolyte Compatibility

While this work proposes a parameter that can be used to approximate the adhesion between any two solid materials, we additionally impose other basic constraints on candidate coating materials in order to provide analysis on plausible materials for solid-state batteries. We choose the following constraints as necessary theoretical criteria for an operational cell. All criteria can be found or calculated using functionalities of the materials project API and pymatgen.^21,22^

**Figure 1:**
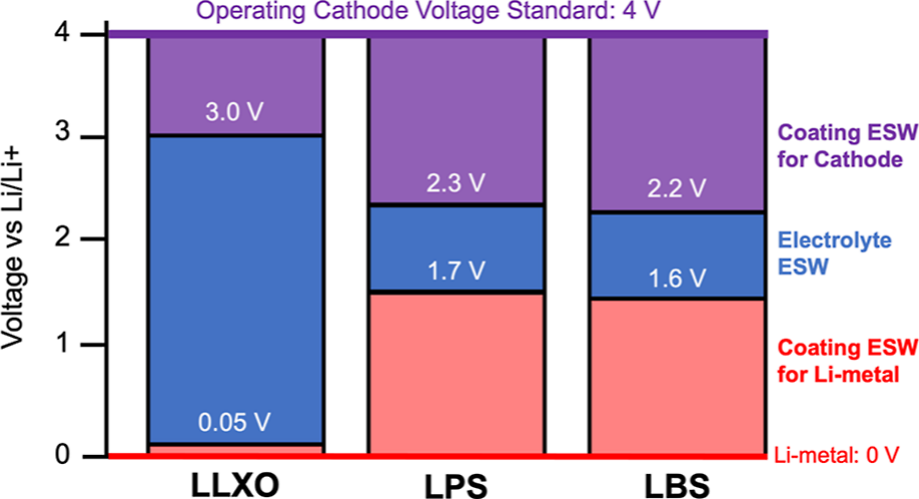
Schematic of the required ESWs of coatings for each of the electrolyte families of interest.

As previously described, we first only consider materials which contain lithium. This is a necessary criterion for coating materials as an extension of the electrolyte functionality to prevent a decrease in capacity of the battery from lithium absorption by the coating material. As an extension of the electrolyte, we ensure that materials have a band gap larger than 2 eV, to prevent electronic conductivity, which would expose the electrolyte to the electrode’s electronic potential that the coating is supposed to be shielding. Placement of the coating within the solid-state battery is briefly discussed in the Supporting Information. We further only consider candidates which lie on the convex hull of their chemical systems, or *E*_hull_ = 0, and materials with elements below number 80, which are constraints regarding stability and ease of synthesizability, respectively. For coating candidates not to hinder the operation of a cell, the ESW of the coating must fully span the ESW of the electrode and overlap with the window of the electrolyte. Using Li/Li+ as the reference material, the Li metal electrode has an operational ESW of 0 V and we take a cathode standard to reach an operational voltage of 4 V.^23−26^ The ESWs of each candidate are computed by constructing the convex hull of the grand potential phase diagram as a function of applied Li chemical potential using methods of Ong et al. (2008) and the phase_diagram module of pymatgen.^27^Figure 1 shows the ESW requirements of coatings for the three electrolyte systems we have chosen based on experimental values discussed in these references for each family: LLXO,^28^ LBS,^29^ and LPS.^30^

In addition to individual materials’ properties, we consider the most basic requirement of two materials at the interface, chemical interfacial stability. The reaction energy between each candidate and its respective electrolyte or electrode are computed by the interface_reactions module in pymatgen.^31^ We take the thermodynamic driving force of each pair of materials to come from the combination of the materials which maximizes the exothermic energy release. This value, *E*_rxn_, acts as an upper bound on the driving force of bulk materials to react when placed within the same system. We screen for materials with a maximum driving force of greater than −0.1 eV between the coating and electrolyte, accounting for kinetic stability,^22^ again only presenting the materials that have the highest viability for experimental success.

Three further properties which are integral to battery operation and production are ionic conductivity, elemental cost, and elemental abundance. Ionic conductivity in electrolyte materials was the paramount barrier for replacing liquid with solid electrolytes, and we incorporate experimentally validated ionic conductivities into our analysis of the identified well-adhering compounds. Elemental cost and elemental abundance have both economic and social effects as seen most notably with cobalt.^5^ As the primary goal of this paper is to present a successful approximation for adhesion between materials, we discuss coatings for each electrolyte based on their performance of adhesion, particularly analyzing any trends in anionic groups of the high-performing coatings.

### 2.3 Construction of the Adhesion Parameter

Historically, adhesion is characterized by the contact angle of a drop of liquid on a solid surface,^16^ with the analogous solid–solid interface described by the same relationship.^32^ The larger the contact angle, the worse the adhesion between the two materials. In solid systems, this contact angle is converted into a relation of surface energies between the two materials in the following expression
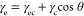
1where γ_e_ and γ_c_ represent the electrolyte and coating surface energies, respectively, and γ_ec_ represents the electrolyte-coating interfacial energy for our system. We approximate “good” adhesion as θ ≤ 90 deg, and a perfectly adhered system would have θ = 0 deg. Within any bulk material, the surface energy could be described by all of the various terminations and Miller orientations that can be created through a unit cell. In our work, we approximate the surface energy of all possible terminations and orientations by DFT. Details of these calculations can be found in the Supporting Information.

Surface energy values have a lower bound of 0, as it always takes energy to break bonds to create a surface. However, interfacial energies can be positive or negative, and the lower (more negative) values represent more stable, and therefore perhaps more likely naturally occurring interfaces. We hypothesize that more stable surfaces are less likely to bond or adhere to other materials. In the derivation below min(γ_*x*_) refers to the most stable termination among all terminations and orientations (up to miller index 1) of material *x*. We utilize min(γ_*x*_) and max(γ_*x*_) to establish our adhesion parameter approximation as a lower bound on adhesion between two solid materials.

We begin by isolating cos θ, and stating its bounds
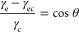
2where −1 ≤ cos θ ≤ 1. The true value of the ratio of surface energies is limited by bounded cases of each individual surface energy. These bounds exist because of the various possible terminations or exposed surfaces of each material.
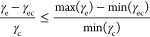
3

We substitute cos θ from eq 2 into eq 3 and isolate γ_ec_ as the variable of interest.
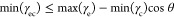
4

In order to achieve the most favorable interfacial interaction, represented by the lowest γ_ec_, we aim to minimize the right side of the equation which will act as an upper bound on min(γ_ec_). To find materials which are best adhered, we use the case of perfect adhesion between two materials θ = 0. For adhesion to be favorable, γ_ec_ < 0, and therefore the goal of this work is to identify materials for which
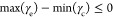
5

The left side of eq 5 will be further referred to as the “adhesion parameter” in units of eV/Å^2^. This provides an approximation for adhesion from values which can be quickly calculated from quantum mechanical methods for solid materials. A detailed description for our approximation of γ is in the Supporting Information.

## 3 Results

**Figure 2:**
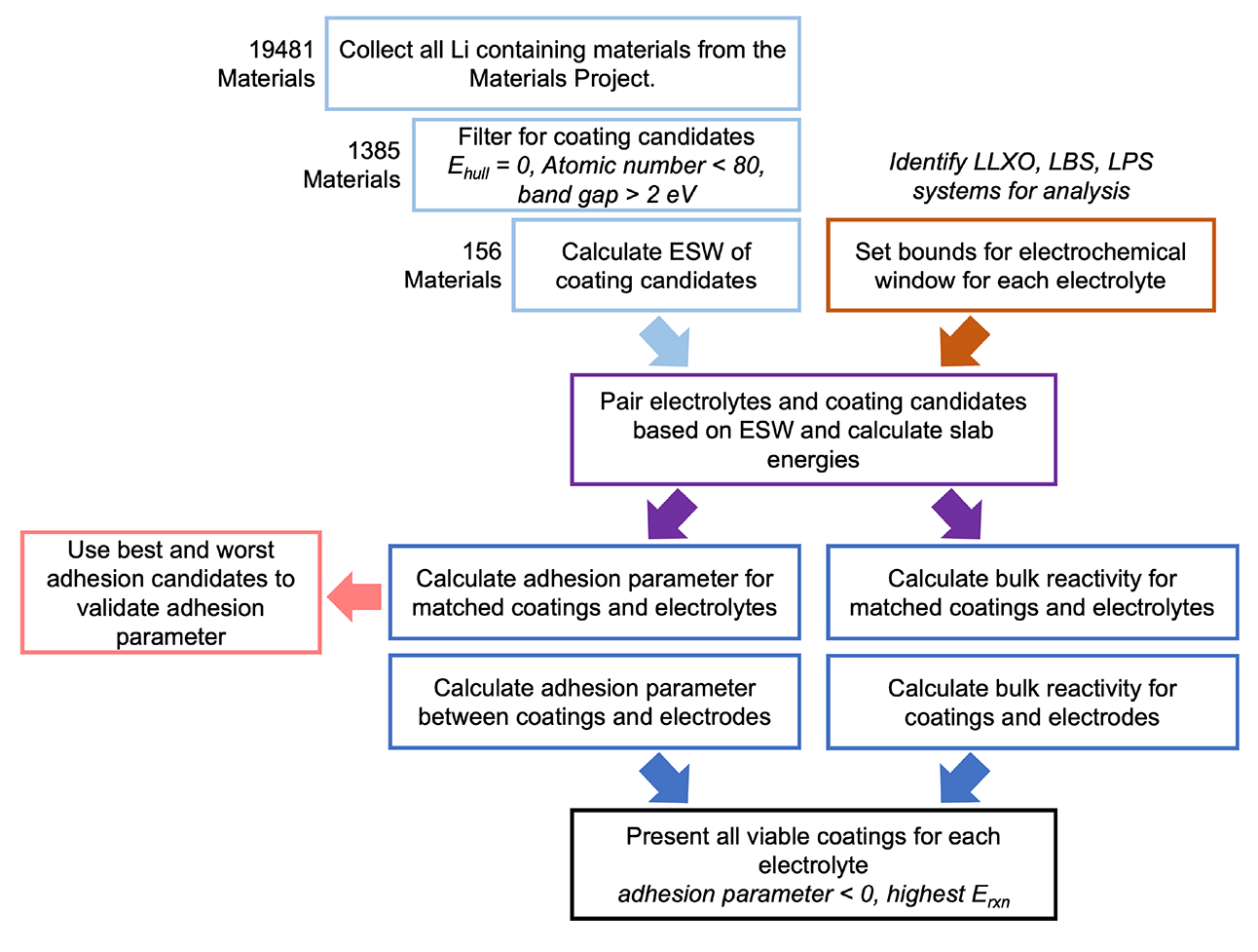
This graphic outlines the order of filters applied and calculations done for our coating candidate data set.

Figure 2 describes the process of data collection, processing, and analysis for this work. The order in which candidate materials were filtered out was chosen to minimize computational expense. We validated the performance of our adhesion parameter against examples of interfacial energies; those specific interfaces and their quantitative results are reported in the Supporting Information. Then the adhesion parameter is calculated on all materials which pass these necessary markers for baseline battery operation and we analyze the materials that could have higher performance in batteries. Additionally, we include the bulk reactivity in our analysis, as described in Section 2.2. The best materials across all electrolytes are discussed in Section 4 of this paper, and we also provide the entire list of candidate coatings for which we calculate a favorable adhesion parameter with each electrolyte in the Supporting Information.

### 3.1 Materials Screening

Beginning from 19481 lithium-containing materials in the materials project, the atomic number, band gap, and *E*_hull_ filters quickly reduce our candidate list to 1385 candidates. After pairing the ESWs, the electrolyte systems have LLXO:36, LBS:11, and LPS:11 for lithium metal side candidates, and LLXO:156, LBS:82, and LPS:93 for cathode side candidates. These results contain 156 unique materials, totaling 945 slab terminations. Fifteen of these materials did not converge during our DFT calculations after ∼2 weeks runtime, likely due to extremely unstable or large surfaces of particular slabs. Our analysis of materials is generalized, and the unconverged materials will not skew our general analysis or prevent us from validating our adhesion parameter approximation as there are other similar materials in the data set from which we can gain insight. Materials for which DFT calculations did not converge are listed in the Supporting Information.

### 3.2 Adhesion Parameter Approximation

**Figure 3:**
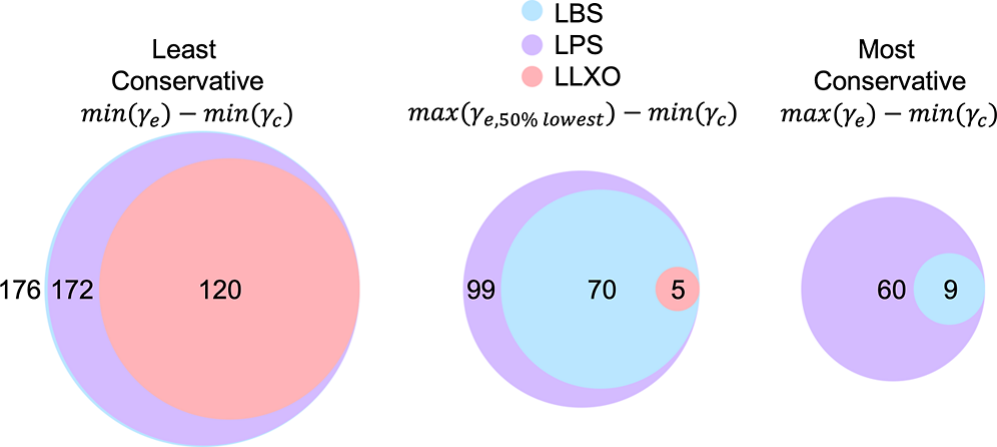
These diagrams show the number of coatings which meet the ESW criteria and have a favorable adhesion parameter with the electrolyte system, for three different levels of conservativeness.

The slab with the lowest surface energy for each coating was chosen to represent γ_c_ in our adhesion parameter approximation. Our calculation of the adhesion between interfaces is conservative, allowing us to narrow in on materials which have the highest chance of wetting to an electrolyte or electrode. The max(γ_e_) value represents the most unstable termination of the electrolyte material, which is unlikely to occur as an exposed surface, both because electrolytes take a polycrystalline form and more stable terminations are more naturally occurring. For this reason, we also analyze two less conservative scenarios. First, we instead use the maximum value of the lowest 50% of surface energies (i.e. the upper bound of the more likely occurring surfaces). Second, we use the most stable termination for all electrolytes, which is the least conservative approximation. The differences in the number of materials which align with each of the bounds for γ_e_ are shown in Figure 3.

**Figure 4:**
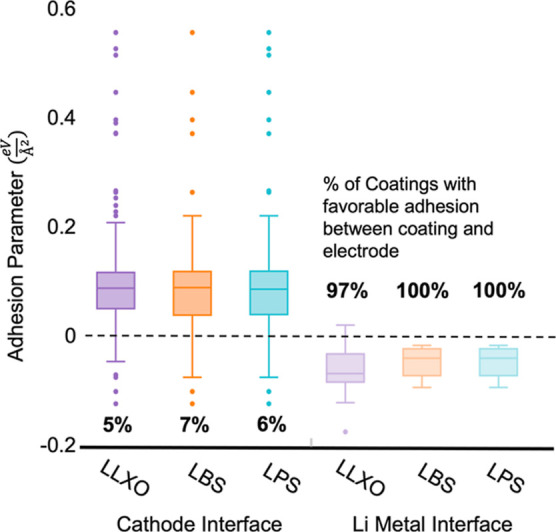
Each box and whisker plot represents the distribution of adhesion parameters at the electrode-coating interface with compatible ESWs and meet stability and band gap constraints for the electrolyte system. This includes coatings which have poor adhesion between the electrolyte and coatings. As defined in Section 2.3, an adhesion parameter less than zero is considered good adhesion.

The results of our adhesion parameter calculations for the electrode-coating interface are shown in Figure 4. We easily reveal the disparity in the number of available coatings which would adhere well to the respective electrode. These distributions demonstrate that the limiting interface with adhesion is not only between the electrolyte and coating for anode coatings but also between the cathode and its coatings. When further considering the electrode-coating adhesion for our final list, the LLXO electrolytes had significantly less well-adhering, nonreactive candidates (5), compared to LBS (33) and LPS (49) systems. The range for means across all electrolytes is very narrow, and the LLXO electrolytes show the lowest (best) adhesion parameter overall. Both Li_10_Ge_2_S_12_ and Li_5_B_7_S_13_ have significantly fewer promising candidates as other electrolytes in their groups, highlighting that small elemental and structural differences can greatly affect the bonding between surfaces. The fact that we identified more than 70 materials that adhere well to sulfide electrolytes shows the importance of this work, in being able to extend the ESWs and allow for more feasible electrode–electrolyte combinations in batteries. Because we are taking a conservative approach with this approximation, it is highly likely that these distributions underestimate adhesion between materials.

### 3.3 Bulk Reactivity and Electrode Analysis

As an added metric for stability between candidate coatings and electrolytes or electrodes, we calculate the bulk reactivity (*E*_rxn_) between pairs of materials, which assesses the thermodynamic driving force for a reaction at the interface. By setting a threshold for *E*_rxn_ in combination with a high adhesion parameter, we identify the most promising materials which should bond at the interface, but not react to consume each other and form byproducts. From the set of well-adhering candidates, the cutoff of −0.1 eV is limiting for some electrolytes. LLTO has no candidates with *E*_rxn_ ≥−0.25 eV, and other electrolytes have only a few candidate materials that we present for discussion. We then repeat adhesion parameter and *E*_rxn_ calculations on coatings with either lithium metal or common cathode materials LiFePO_4_, LiCoO_2_, LiMnO_2_, and LiNiO_2_. There are very few coatings which can withstand all of these tests to be considered a promising candidate. For our analysis of top materials, we focus on materials which were found to be most promising for each electrolyte group. In the Supporting Information, we present all materials with a negative adhesion parameter along with their *E*_rxn_ values and data for interfacing with cathodes and lithium metal.

## 4 Discussion

All materials for which coating candidates had a negative adhesion parameter and an *E*_rxn_ > −0.1 eV with both an electrolyte and at least one electrode are shown in the Supporting Information; we will refer to this set as “favorable candidate coatings”. Most notable is that the limiting factor on the anode coatings is the adhesion between the electrolyte and the coating. We hypothesize that bulk and surface stability trend similarly; therefore, coatings which are more electrochemically stable against Li have more stable surfaces and bond less strongly with the electrolyte. Coating materials listed in the Supporting Information intended for use with the Li metal electrode were only predicted to be well-adhering to Li metal, not the respective electrolyte, but have an *E*_rxn_ = 0 eV with Li metal and meet our other requirements. The calculated adhesion between coatings and electrodes was used for analysis and pairing down to our best candidates, but the values are not listed explicitly, similar to our other quantitative screening constraints. For this work, we focus on the materials which meet all requirements outlined in our methods. We additionally analyze these materials through the lens of ionic conductivity, another necessary metric for high performance of these coating materials. With the electrolyte materials all exhibiting ionic conductivities >10^–4^ S/cm, the two barriers to a fast conducting system are the interfacial transfer between electrolyte-coating and electrode-coating and the ionic conductivity of the coating. The adhesion parameter is designed to mitigate the former; therefore, we aim to find coatings with ionic conductivities >10^–4^ S/cm as well, to mitigate the latter. In this discussion we will refer to ionic conductivities from literature when available.

Across the three electrolyte systems, sulfides show candidates with more negative (more favorable) adhesion parameters, explained by the upper bounded approximation in the adhesion parameter. If the electrolyte surface used for the adhesion parameter calculation are much more unstable than the most naturally occurring terminations, the electrolyte will appear to have worse adhesion with all possible coating candidates. There are likely more coatings which would adhere well to the electrolyte systems, but with this work we aim to present only our most viable candidates. Because a coating layer is necessary for the stability of the electrolyte/electrode interface, we believe it is worth investigating chemical optimization of the discussed compounds to increase the conductivity, as they meet all other criteria. Below we highlight key insights from our results including: effect of anion group on ESW, oxidation state and covalency effects on adhesion, stoichiometric effects within chemical systems, and current literature on our most promising candidates.

### 4.1 Anion Composition of Materials with Good Adhesion

**Figure 5:**
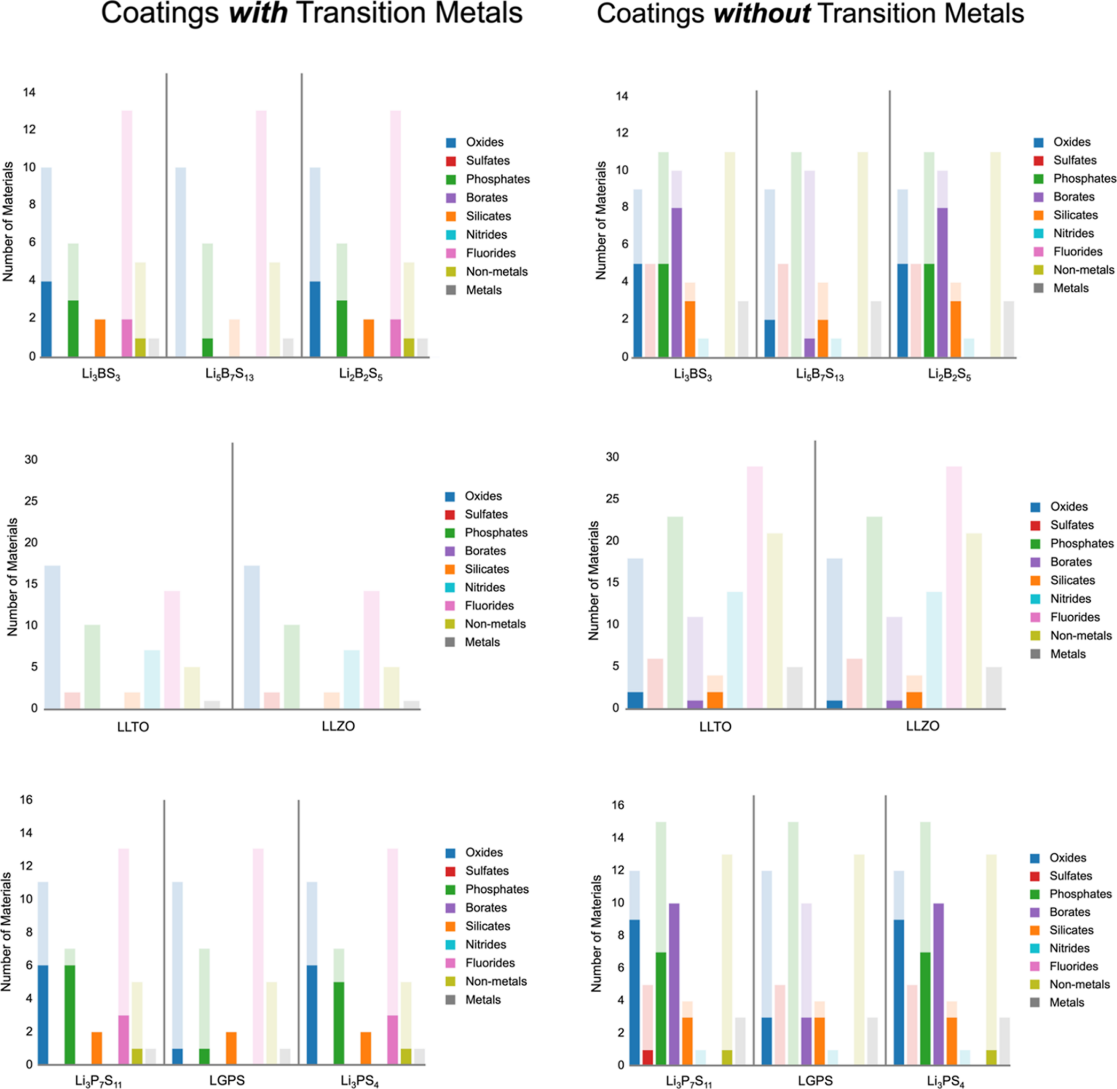
Plots show the coating candidates and their resulting performance sorted by the anionic element or group in the material. The lighter background shows the number of candidates that passed the ESW and elemental screening, and the solid foreground shows the number of materials that have good adhesion with that particular electrolyte.

We labeled materials by common anion groups (oxides, sulfides, phosphates, borates, silicates, nitrides, fluorides, non-metals, and metals) to better understand the characteristics of coatings more likely to bond well with electrolytes. The background bars in Figure 5 show the distribution of anion types for materials which had compatible ESWs with each electrolyte group and passed the metrics outlined in Section 2.2. The most notable difference between electrolyte groups is that there are zero non-metal, sulfate or phosphate coatings available to LLXO systems, which are available to LPS and LBS groups. The minimum, median, and maximum cleavage energy for each system is listed in Table 1, showing vastly differing distributions of termination cleavage energies. The median values for LLTO and LLZO (and Li_5_B_7_S_13_) are all significantly higher, representing more unstable surfaces. This approximation is an upper bound on what would be an energy weighted average of all terminations; therefore, there would likely be more well-adhering coatings. However, our screening approach allows only extremely stable coatings to achieve all of our screening benchmarks, increasing the likelihood for success in further development.

**Table 1:** Listed are the Distribution Descriptors of Cleavage Energies for Each Electrolyte System, Showing the Varying Distributions Across Electrolytes

	cleavage energies (eV/Å^2^)		
electrolyte	minimum	median	maximum
LLZO	0.093	0.188	0.279
LLTO	0.036	0.179	0.289
LiB_3_S_3_	0.023	0.069	0.137
Li_5_B_7_S_13_	0.095	0.167	0.241
Li_2_B_2_S_5_	0.008	0.074	0.151
Li_3_PS_4_	0.017	0.056	0.072
Li_3_P_7_S_11_	0.011	0.053	0.086
Li_10_GeP_2_S_12_	0.037	0.064	0.122

From the plots in Figure 5, we can see the effect that stoichiometry and crystal structure have on adhesion. Li_5_B_7_S_13_ has significantly less favorable adhesion candidates than Li_3_BS_3_ and Li_2_B_2_S_5_, which means the available electrolyte terminations have higher surface energies in Li_5_B_7_S_13_. Li_5_B_7_S_13_ has lower Li/B and Li/S ratios, suggesting that boron and sulfur are less likely to bond when in contact with other surfaces. We see a related trend in the LPS family, where Li_10_GeP_2_S_12_ exhibits less candidates for adhesion, even though it has a higher lithium content. Here, the germanium atom likely destabilizes the surface, similarly to the transition metals causing unstable surfaces in the LLXO system. Oxides and silicates perform well across all compounds, which could ease the process for experimental investigation as these families are typically environmentally stable (i.e., in air and water).

A few anion groups were not favorable for most systems. Nitrides cannot meet the larger ESW requirements for sulfur electrolytes; hence, there were very few candidates for which we could approximate adhesion.^33^ There were no metal compounds with favorable adhesion, which is surprising considering the work done on LiH as a successful coating. It was even shown that LiH was able to decrease the presence of Li dendrites in an LiMg anode, due to the inherent electric field between LiH and LiMg.^34^ However, our adhesion parameter favors high bonding energies between two materials, and it seems that lithium-containing metals are too similar to Li metal to meet our criteria. Additionally, borates only have favorable adhesion in compounds which don’t also have a transition metal, and fluorides only have favorable adhesion in compounds including a transition metal. A highly electronegative element such as fluorine would be more reactive and stabilizes a transition metal ion, as compared to a borate group.

The LLXO systems only adhering with silicate, borate, or oxide coatings speak to the stability of those coating systems. Silicates (e.g. SiO_4_) and borates (e.g. BO_4_) are metalloid polyanionic systems, as opposed to the phosphates and sulfates. The electronegativity difference between oxygen and boron or silicon creates more tightly bound anion groups, which then create a host lattice in which the Li resides. Because of the covalent character within the non-metalloid anionic groups, terminations with exposed atoms remain stable at many coordinations. We hypothesize that due to charge sharing, there is a lower probability of exposed unshared electrons, increasing the stability of a termination for non-metalloid groups. The non-oxygen anions, phosphorous and sulfur, have more flexible oxidation states than oxygen meaning they can perhaps be stabilized more readily in the presence of a charge compensating transition metal, which is also exhibited in our results.

### 4.2 Cathode Coatings

The following coatings had a favorable adhesion parameter with both their respective electrolyte systems and the representative cathode coatings, in addition to an *E*_rxn_ < 0.1 eV. LiAl_5_O_8_ and LiAlSiO_4_ are the top two candidates across all systems. LiAl_5_O_8_ has been extensively studied in battery operations across various morphologies. The flexibility of synthesis methods for this material into nanowire composites, sintered thin films, and sol–gel coatings with distinct electrochemical improvements gives a wide space for optimization of this compound.^35−37^ As a nickel–manganese–cobalt cathode coating, LiAl_5_O_8_ was found to increase the coulombic activity and capacity retention with a thin 3 nm film.^35^ Though its independent Li-ion conductivity was found to be only ∼10^–6^ S/cm, its ability to be cast into films under 10 nm can lessen the impact of its lower ionic conductivity.^38^ LiAl_5_O_8_ also limits side reactions and chemical degradation of the cathode material. Wang et al. additionally found LiAl_5_O_8_ to suppress Li metal dendrites in polymer solid-state batteries.^37^ Its potential for optimization of other electrochemical metrics makes LiAl_5_O_8_ extremely promising for further adaptation. LiAlSiO_4_ is similarly viable for further investigation, as it was shown to improve capacity retention and it has been verified for synthesis and assembly into batteries experimentally.^39^ The synthesis methods take advantage of the glassy nature of this material, whose amorphous phase allows for increased ionic conductivity.^40^ LiAlSiO_4_ can be prepared and coated using more simple solution and drying techniques, which makes this appealing to adopt further. There is room for optimization of Li content and cathode particle size, as there have been investigations to optimize weight ratios of the coatings as well as increased ionic conductivity with thin film morphologies.^41,42^

LLXO-specific candidates are only slight deviations of the universal candidates LiAl_5_O_8_ and LiAlSiO_4_. The ordered phases of LiGaSiO_4_ and LiAlGeO_4_ have trigonal symmetry, though are not layered materials like the trigonal cathodes. This is likely the cause for the decrease in ionic conductivity in our coating candidates, which only reach favorable ionic conductivities approaching 1000 K.^43,44^ However, an extreme improvement up to ∼10^–5^ S/cm was found in solid solutions of Li_4_SiO_4_ and Li_5_GaSi_2_O_8_, which gives hope to a possible similar improvement with the mixture of the candidate coatings presented in this work.^45^ With these structures similar to presented candidate LiAlSiO_4_, it would be worth investigating glassy phases of these coatings for increased ionic conductivity.

In both LPS and LBS electrolyte systems, Li_2_B_6_O_9_F_2_ was found as the highest performing coating unique to sulfide electrolyte systems. This material has been identified as a coating candidate in previous computational studies, which serves as validation of the methods in this work.^46^ However, the experimental ionic conductivity was measured at ∼10^–10^ S/cm, making it a likely barrier for conduction between cathodes and electrolytes.^47^ A candidate unique to only the LPS system is . The effects of substituting larger potassium or cesium atoms for lithium may have overall negative effects on the ionic conductivity, given the larger size of the atoms.

### 4.3 Li Metal Coatings

Most stable coatings for sulfide systems are found to adhere well to Li metal with an *E*_rxn_ = 0 eV, but they have a less favorable adhesion to the electrolytes. Combined incorporation of candidates LiCl and LiBr systems has recently been investigated to increase their ionic conductivity, as LiCl alone has been shown to have insufficient ionic conductivity (∼10^–6^ S/cm).^48−50^ Previous work by Lutz et al. has specifically investigated lithium chlorides for the purpose of coatings, though candidate CsLiCl_2_ has not been specifically investigated.^100^ Across a much wider array of cathodes than was screened in this work, the chlorides showed to have an *E*_rxn_ < ∼100 meV, many we believe can be kinetically stabilized. In addition, all of the explored ternary chlorides have a room-temperature conductivity of >10^–4^ S/cm, a promising trend for the family of our new compounds. The only candidate presented for LLXO systems, Li_5_SiN_3_, has previously been investigated as both a cathode and solid-electrolyte material.^51,52^ This is exciting as the material is synthesizable and able to be assembled into a test cell.

If we lowered the 2 eV band gap requirement, we find Li_4_CrFe_3_O_8_ (band gap = 1.85 eV) is the only coating in our entire screening process to adhere well to an electrolyte for a Li metal coating and it is within the LBS system. Lithium chromium ferrite is a similar compound which has been studied for its magnetic properties.^53,54^ This is a layered oxide, similar to the structure of common cathodes, but with iron and chromium in their 3+ oxidation state. We believe that there will not be a large driving force for redox activity, similar to zirconium in LLZO. Other iron-oxide stoichiometries have been studied for electrode materials. It was found that doping α-Fe_2_O_3_ with chromium improved the rate performance and lithium ionic conductivity, while chromium-doped γ-Fe_2_O_3_ improved the cycling performance.^55,56^

## 5 Conclusions

This work was directed to complement the screening literature for solid-state battery materials, by investigating thermodynamic interactions at the interface of materials. Experimentally measuring surface energies by contact angle requires extreme surface control, and time-consuming computational ionic relaxations of numerous surface terminations makes the determination of interfacial energies infeasible even for a small number of systems.^57^ We proposed and examined a quick to compute metric for adhesion between two crystalline materials which allows for screening on large scales. The adhesion parameter approach in which we look at limits of the adhesion energy is specifically effective to address the complex nature of polycrystalline interfaces without computing the vast number of combinatorial interface configurations. In utilizing this parameter to screen for materials, we were able to find materials which account for ionic conductivity, stability, and adhesion at interfaces in solid-state batteries. With our surface-dependent metric, we can extend our evaluation of candidates to more accurately identify and recommend materials whose bulk metrics have been corroborated by previous experiments. The short coating candidate list for electrolyte systems after screening through more than 19,000 materials demonstrates the difficulty in finding materials that meet all requirements. The sulfide electrolyte coating candidates showed promise when looking at attainable glassy structures, which can increase the ionic conductivity over crystalline forms. The LLXO system had many candidates of various oxides, which upon further investigation could be combined to maximize ionic conductivity. Many of the presented coatings have been experimentally synthesized and assembled into batteries as electrolyte coatings, putting them at the optimization stage for further investigation, accelerating their commercialization viability. Specifically, LiAl_5_O_8_ and LiAlSiO_4_ are top coating candidates across all electrolyte systems. Their flexible morphologies allow for more simple synthesis methods and diverse avenues for optimization such as Li content. We believe through better adhesion at the atomic level, solid-state batteries can achieve lower interfacial resistance and avoid mechanical delamination. The materials highlighted in this work can serve as a platform for coating optimization.

## Supporting Information Available

Description of cleavage energy calculations, description of coating placement within the battery, and full list of satisfactory candidates (PDF)
