# Supplementary material for: Electrolyte Coatings for High Adhesion Interfaces in Solid-State Batteries from First Principles
Source: ACS Appl Mater Interfaces. 2023 Sep 8;15(37):44394–403. doi: 10.1021/acsami.3c04452 (PMC10520915; doi:10.1021/acsami.3c04452)
Supplement: Supplementary file 1 — am3c04452_si_001.pdf [file am3c04452_si_001.pdf]

Supporting Information

Electrolyte Coatings for High Adhesion Interfaces in Solid-state Batteries from First Principles

Brandi Ransom,† Akash Ramdas,† Eder Lomeli,† Jad Fidawi,† Austin Sendek,†,‡ Tom Devereaux,†,¶ Evan J. Reed,† and Peter Schindler*,§

†*Department of Materials Science and Engineering, Stanford University, Stanford, California, 94305, United States*

‡*Aionics, Inc., Palo Alto, California, 94301, United States*

¶*Stanford Institute for Materials and Energy Sciences, Stanford University, Stanford, California, 94305, United States*

§*Department of Mechanical and Industrial Engineering, Northeastern University, Boston, Massachusetts, 02115, United States*

E-mail: p.schindler@northeastern.edu

Phone: 650-248-1066

Cleavage Energy Calculations

The adhesion parameter approach in which we look at limits of the adhesion energy is specifically effective to address the complex nature of polycrystalline interfaces without computing the vast number of combinatorial interface configurations. The adhesion parameter was calculated between each candidate and its respective electrolyte pairing for every lithium containing material in the Materials project data base that met all constraints described in section 2.2. Cleavage energies were implemented as surface energies (γ) in our adhesion parameter calculation. They require

calculations on both the bulk material and isolated slabs created from different possible Miller orientations and terminations where stoichiometry can be sustained. The cleavage energy can be calculated from the following equation:

$$E_{cleavage} = \frac{E_{slab} - n_{bulk} * E_{bulk}}{2 * A_{slab}} \quad \text{(S1)}$$

$E_{cleavage}$ is calculated for all slabs of a particular material. E_x represents energies from self-consistent field calculations for both slab and bulk geometries. n_{bulk} is the number of bulk unit cells in the slab and A_{slab} represents the surface area of the slab normal to the surface. The cleavage energy is defined, where A and B represent two surfaces of a slab, by

$$E_{cleavage} = \frac{\gamma_A + \gamma_B}{2} \quad \text{(S2)}$$

therefore ...

$$2 * E_{cleavage} = \gamma_A + \gamma_B. \quad \text{(S3)}$$

Because $\gamma_A \geq 0$ and $\gamma_B \geq 0$,

$$2 * E_{cleavage} \geq \gamma_A, \gamma_B. \quad \text{(S4)}$$

and we use $2 * E_{cleavage}$ as the upper bound for any γ for non-symmetric slabs (i.e., where top and bottom surfaces are not equivalent).

If the slab expresses symmetry of a mirror or glide plane parallel to the surface, or a 2D rotation normal to the surface, the cleavage energy exactly equals the surface energy. Calculating the exact surface energies for all non-symmetric terminations requires surface pourbaix phase diagrams and chemical potentials for all relevant elements, which is cumbersome. Therefore, we choose to use cleavage energies as an upper bound which suffices in our use case. As the adhesion parameter inequality would be most easily satisfied by a low $max(\gamma_e)$ and high $min(\gamma_c)$, we want to use these to bound our approximation for electrolyte slab energies. To adhere to this constraint, $min(\gamma)$ values from cleavage energies for the electrolyte materials include considering the lower surface energies

from symmetric slabs. However for coating materials we calculate symmetric slabs with the same 2x upper bound factor on cleavage energies, in order to respect the lower bound approximation on adhesion we are calculating.

For each bulk material, surfaces up to a Miller index of 1 were generated using pymatgen's surface module.[1–3] Each surface generally has more than one unique surface termination. We developed an algorithm to ensure that we generate all possible *unique* terminations based on the local environment of surface atoms. Details of this algorithm are described in our previous work.[4] All slabs generated have a minimum thickness of 10 Å and 15 Å of vacuum between periodic slab repetitions in the *c*-direction to preclude interactions between periodic images and based on previous convergence investigation of slab energies by Schindler et al.[5] Self Consistent Field DFT calculations were performed using the Projector Augmented Wave pseudopotential implementation of the Vienna Ab Initio Simulation Package, version 5.4.1. In the DFT calculations, electron exchange and correlation effects are described by the Generalized Gradient Approximation (GGA) functional of PBE. Wave functions are expanded in a plane-wave basis set with a kinetic energy cutoff of 520 eV using gaussian smearing of 200 meV, and electronic relaxation convergence threshold was 0.1 meV. The dipole correction was not used as we find this functionality specifically important in work functions of materials, which we do not consider in this work. Spin polarization was not used due to the increased compute time necessary for these calculations, and because the error between spin-polarized energies is about an order of magnitude lower than the total energy.[6] We made sure to calculate the bulk reference energy on re-oriented structures (same orientation as the surface) that ensures convergence of the surface energy for slabs with fewer atomic planes (due to the matching k-point sampling between slab and bulk reference).[7] The energy results of these calculations were input as E_{slab} and E_{bulk} in equation S1.

Materials for which these DFT calculations did not converge are: Li_3GaF_6, $K_2Li_3B(P_2O_7)_2$, $Rb_2Li_3B(P_2O_7)_2$, $CsLi(PO_3)_2$, $Li_2Ge(S_2O_7)_3$, $KLi_3Ca_7Ti_2Si_{12}(O_{18}F)_2$, $Na_3Li_3Sc_2F_{12}$, $LiVZnO_4$, Li_2CrO_4, Li_5TiN_3, $KLiMoO_4$, $LiZnAsO_4$, $Li_7La_3Hf_2O_{12}$, $LiZnPO_4$,Li_2MoO_4.

Interfacial Energy Calculations and Adhesion Parameter Benchmarking

In order to benchmark the protocol of substituting cleavage energies for surface energies described above, we computed the interfacial energies for best and worst adhering systems as determined by our adhesion parameter. These were calculated by DFT for both the surfaces and the bulk structures of each material, and using the following equations for electrolyte or coating slabs, and electrolyte/coating slabs, respectively.

$$\gamma_{(e/c)} = \frac{E_{(e/c),slab} - n_{(e/c)} E_{(e/c),bulk}}{2 * SA_{(e/c)}} \tag{S5}$$

$$\gamma_{ec} = \frac{E_{ec,slab} - n_e E_{e,bulk} - n_c E_{c,bulk}}{2 * SA_{ec}} \tag{S6}$$

Where n_x corresponds to the number of unit cells of materials contained in the slab. The values computed are then used to calculate the interfacial energy, which is negative if there is favorable adhesion.

$$E_{int}/\text{Å}^2 = 2 * (\gamma_{ec} - \gamma_e - \gamma_c) \tag{S7}$$

Six electrolyte/coating systems and four Li metal/coating systems were considered to benchmark the adhesion parameter by considering the energetics of fully optimized interfaces and surfaces. A selection of the best and worst adhering interfaces from the Li metal-coating, LBS, LPS, and LLXO systems were chosen. Cathode/coating interfaces were not considered during benchmarking because cathodes are ceramics similar to the electrolyte materials and hence benchmarking the electrolyte/coating system is expected to generalize to cathode/coating-type interfaces. The calculations to obtain interfacial energies of each material involved full ionic-relaxations of slab terminations (γ_e, γ_c), as well as ionic-relaxations of the interfaces (γ_{ec}). Due to artifacts from creating interfaces with small area slabs, we report the interfaces which were able to reach convergence in under 1 month of computing. These interfaces and their benchmarked values are shown in

table S1.

Table S1 This table shows the quantitative values of benchmarking our adhesion parameter with interfacial energies on select interfaces. Positive values represent poorly adhering interfaces, and negative values represent well-adhering interfaces.

Coating	Electrolyte or Li metal	Interfacial Energy (eV/Å^2)	Adhesion Parameter (eV/Å^2)
$CsLiF_2$	$Li_7La_3Zr_2O_{12}$	-0.68	0.78
$LiP(HO_2)_2$	$Li_{10}Ge(PS_6)_2$	-0.58	0.49
$LiGaCl_4$	$Li_5B_7S_{13}$	-0.08	0.16
$LiAl_5O_8$	$Li_7La_3Zr_2O_{12}$	-1.02	0.01
$LiAl_5O_8$	$Li_7P_3S_{11}$	-0.68	-0.13
$LiTaSiO_5$	$Li_2B_2S_5$	-0.42	-0.05
$LiCaAlN_2$	Li metal	-0.30	-0.11
$LiTbO_2$	Li metal	-0.26	-0.10
$LiCa_4(BN_2)_3$	Li metal	-0.22	-0.10
LiH	Li metal	0.20	0.02

Table S2 This confusion matrix represents the performance of the approximated adhesion parameter. True interfacial energies were calculated from fully optimized geometries of the interfaces and surfaces.

		True Interfacial Energy	
		negative	positive
Predicted Adhesion Parameter	negative	5	0
	positive	4	1

A confusion matrix of the performance of benchmarked interface calculations vs. the approximated adhesion parameter is show in Table S2. All of the Li metal/coating interfaces were correctly classified with the adhesion binary using the approximation. While the adhesion parameter misclassified 4/9 materials as having poor adhesion (recall: 55.6%), the precision of the method is 100% for the 10 samples tested (all materials labeled as well-adhering by the adhesion parameter were validated to be well adhering). It's worth pointing out that calculating the adhesion parameter is about 10^3 times faster (depending on the system) than calculating the full interface energy. Therefore the adhesion parameter in ceramic-ceramic systems works well as a screening tool in narrowing the materials space to promising candidates for further exploration.

Full list of Satisfactory Candidates

Here we present the full list of coating candidates which had a negative adhesion parameter and an $E_{rxn} > -0.1\ eV$ when interfaced with the electrolytes. All candidates also had a negative adhesion parameter and an $E_{rxn} > -0.1\ eV$ for their interface with cathodes. Candidates marked with $*$ had a positive (unfavorable) adhesion parameter ≤ 0.05 with electrolytes, but did have an $E_{rxn} = 0\ eV$ and negative adhesion parameter with Li metal. There was only a single candidate with a favorable adhesion parameter and $E_{rxn} = 0\ eV$ with Li metal: $Li_4CrFe_3O_8$ in interface with the LBS system, though its band gap was slightly below our threshold. We include the group of materials with the lowest E_{rxn} value for $Li_5La_3Ta_2O_{12}$ because no coatings had an $E_{rxn} > -0.1\ eV$. The calculated adhesion between coatings and electrodes was used for analysis and pairing down to our best candidates, but the values are not listed explicitly, similar to our other quantitative screening constraints.

Table S3 This table shows viable coating candidates for the LLXO system.

**LLXO System**				
	$Li_5La_3Ta_2O_{12}$		$Li_7La_3Zr_2O_{12}$	
coating	adhesion parameter (eV/Å^2)	E_{rxn} (eV)	adhesion parameter (eV/Å^2)	E_{rxn} (eV)
$LiGaSiO_4$	-0.08	-0.29	-0.07	-0.07
$LiAlGeO_4$	-0.06	-0.29	-0.05	-0.07
$LiAlSiO_4$	-0.03	-0.29	-0.02	-0.06
$LiGeBO_4$	-0.03	-0.29	-0.02	-0.08
$LiAl_5O_8$	-0.00	-0.29		
$*Li_5SiN_3$	0.01	-0.29	0.02	-0.05

Table S4 This table shows viable coating candidates for the LBS system.

LBS System						
	Li_3BS_3		$Li_2B_2S_5$		$Li_5B_7S_{13}$	
coating	adhesion parameter (eV/Å^2)	E_{rxn} (eV)	adhesion parameter (eV/Å^2)	E_{rxn} (eV)	adhesion parameter (eV/Å^2)	E_{rxn} (eV)
$Li_4CrFe_3O_8$	**-0.03**	**0**	**-0.03**	**0**		
$LiAlSiO_4$	-0.14	-0.02	-0.14	-0.03	-0.04	-0.03
$LiAl_5O_8$	-0.11	-0.04	-0.11	-0.06	-0.02	-0.05
$Li_2B_6O_9F_2$	-0.09	-0.02	-0.09	-0.01		
$*Li_2S$	0.03	0.00	0.03	0.00	0.13	0.00
$*Li_2Se$	0.04	0.00	0.03	-0.02	0.13	0.00
$*Li_2Te$	0.04	-0.01	0.04	-0.08	0.14	-0.01
$*LiCl$	0.05	0.00	0.05	0.00	0.15	0.00
$*LiF$	0.05	0.00	0.05	0.00	0.15	0.00

Table S5 This table shows viable coating candidates for the LPS system.

LPS System						
	$Li_7P_3S_{11}$		Li_3PS_4		$Li_{10}GeP_2S_{12}$	
coating	adhesion parameter (eV/Å^2)	E_{rxn} (eV)	adhesion parameter (eV/Å^2)	E_{rxn} (eV)	adhesion parameter (eV/Å^2)	E_{rxn} (eV)
$LiAlSiO_4$	-0.15	-0.03	-0.15	0.00	-0.09	-0.02
$LiAl_5O_8$	-0.13	-0.03	-0.12	0.00	-0.07	-0.02
$K_2LiTa_6(PO_8)_3$	-0.12	-0.06	-0.12	-0.05	-0.06	-0.06
$Li_2B_6O_9F_2$	-0.11	-0.03	-0.10	0.00	-0.05	-0.02
$*Li_2S$	0.01	-0.05	0.02	0.00	0.07	-0.02
$*Li_2Se$	0.02	-0.04	0.02	0.00	0.08	-0.02
$*Li_2CN_2$	0.02	-0.08	0.03	-0.06	0.09	-0.06
$*Li_2Te$	0.03	-0.08	0.03	-0.05	0.09	-0.05
$*LiF$	0.03	-0.03	0.04	0.00	0.09	-0.02
$*LiCl$	0.03	-0.03	0.04	0.00	0.10	-0.02
$*LiBr$	0.04	-0.03	0.04	0.00	0.10	-0.02
$*LiI$	0.04	-0.03	0.05	0.00	0.10	-0.02
$*CsLiCl_2$	0.04	-0.04	0.05	0.00	0.10	-0.02
$*KLiTe$	0.04	-0.09	0.05	-0.05	0.10	-0.06

Coating placement within the Battery

Together with the electrolyte, the coatings help span the electrochemical window between the cathode and the anode, as neither the electrolyte nor the coatings can do it alone. Here we assume the model of a solid-state battery depicted in figure S1, adapted from Hatzell et al's discussion on manufacturing coatings on solid-state batteries.[8]

Because our coatings are an extension of the electrolyte, they do not conduct electronically, meaning we need to intentionally place the coating to not interfere with the electronic pathways created by connecting cathode material. Because of this we imagine the electrolyte materials being coated before they undergo processing or combination with the cathode material. With sufficient coating ionic conductivity, the combination of coating and electrolyte extends the operational electrochemical window of the battery.

The coatings between the anode and solid electrolyte can be applied as a layer.

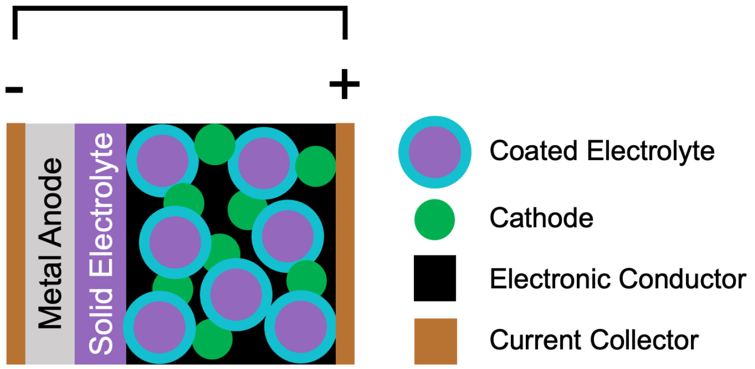

Figure S1 Diagram of solid-state battery.

Notes and references

(1) Jain, A.; Ong, S. P.; Hautier, G.; Chen, W.; Richards, W. D.; Dacek, S.; Cholia, S.; Gunter, D.; Skinner, D.; Ceder, G.; et al., Commentary: The Materials Project: A Materials Genome Approach to Accelerating Materials Innovation. *APL Materials* **2013**, *1*, 011002.

(2) Tran, R.; Xu, Z.; Radhakrishnan, B.; Winston, D.; Sun, W.; Persson, K. A.; Ong, S. P. Surface Energies of Elemental Crystals. *Scientific Data* **2016**, *3*.

(3) Tran, R.; Li, X.-G.; Montoya, J. H.; Winston, D.; Persson, K. A.; Ong, S. P. Anisotropic work Function of Elemental Crystals. *Surface Science* **2019**, *687*, 48–55.

(4) Schindler, P.; Antoniuk, E. R.; Cheon, G.; Zhu, Y.; Reed, E. J. Discovery of Materials with Extreme Work Functions by High-throughput Density Functional Theory and Machine Learning. *arXiv:2011.10905* **2020**, Submitted on Nov 22nd, 2020, https://doi.org/10.48550/arXiv.2011.10905.

(5) Schindler, P.; Riley, D. C.; Bargatin, I.; Sahasrabuddhe, K.; Schwede, J. W.; Sun, S.; Pianetta, P.; Shen, Z.-X.; Howe, R. T.; Melosh, N. A. Surface Photovoltage-Induced Ultralow Work Function Material for Thermionic Energy Converters. *ACS Energy Letters* **2019**, *4*, 2436–2443, PMID: 31633034.

(6) Yu, H.; Sun, J.; Heine, T. Predicting Magnetic Coupling and Spin-Polarization Energy in Triangulene Analogues. *Journal of Chemical Theory and Computation* **0**, *0*, null, PMID: 37263582.

(7) Sun, W.; Ceder, G. Efficient creation and convergence of surface slabs. *Surface Science* **2013**, *617*, 53–59.

(8) Hatzell, K. B.; Zheng, Y. Prospects on Large-scale Manufacturing of Solid State Batteries. *MRS Energy amp; Sustainability* **2021**, *8*, 33–39.
